# A SCID mouse-human lung xenograft model of SARS-CoV-2 infection

**DOI:** 10.7150/thno.58321

**Published:** 2021-05-03

**Authors:** Wenkun Fu, Wei Wang, Lunzhi Yuan, Yanzhen Lin, Xiumin Huang, Rirong Chen, Minping Cai, Che Liu, Liqiang Chen, Ming Zhou, Kun Wu, Huan Zhao, Dequan Pan, Jian Ma, Junping Hong, Bingke Zhai, Yali Zhang, Zhibo Kong, Yingbin Wang, Yixin Chen, Quan Yuan, Huachen Zhu, Tong Cheng, Yi Guan, Ningshao Xia

**Affiliations:** 1 State Key Laboratory of Molecular Vaccinology and Molecular Diagnostics, National Institute of Diagnostics and Vaccine Development in Infectious Diseases, School of Life Sciences, School of Public Health, Xiamen University, Xiamen 361102, Fujian, P. R. China.; 2 Department of Obstetrics and Gynecology, Zhongshan Hospital, Xiamen University, Xiamen 361004, P. R. China.; 3 State Key Laboratory of Emerging Infectious Diseases, The University of Hong Kong, Hong Kong, P. R. China.; 4 Joint Institute of Virology (Shantou University and The University of Hong Kong), Guangdong-Hongkong Joint Laboratory of Emerging Infectious Diseases, Shantou University, Shantou, P. R. China.; 5 Research Unit of Frontier Technology of Structural Vaccinology, Chinese Academy of Medical Sciences, Xiamen 361102, Fujian, P. R. China.

**Keywords:** SARS-CoV-2, infection, human lung, xenograft, humanized mouse model

## Abstract

SARS-CoV-2 infection, which is responsible for the current COVID-19 pandemic, can cause life-threatening pneumonia, respiratory failure and even death. Characterizing SARS-CoV-2 pathogenesis in primary human target cells and tissues is crucial for developing vaccines and therapeutics. However, given the limited access to clinical samples from COVID-19 patients, there is a pressing need for *in vitro/in vivo* models to investigate authentic SARS-CoV-2 infection in primary human lung cells or tissues with mature structures. The present study was designed to evaluate a humanized mouse model carrying human lung xenografts for SARS-CoV-2 infection* in vivo*.

**Methods:** Human fetal lung tissue surgically grafted under the dorsal skin of SCID mice were assessed for growth and development after 8 weeks. Following SARS-CoV-2 inoculation into the differentiated lung xenografts, viral replication, cell-type tropism and histopathology of SARS-CoV-2 infection, and local cytokine/chemokine expression were determined over a 6-day period. The effect of IFN-α treatment against SARS-CoV-2 infection was tested in the lung xenografts.

**Results:** Human lung xenografts expanded and developed mature structures closely resembling normal human lung. SARS-CoV-2 replicated and spread efficiently in the lung xenografts with the epithelial cells as the main target, caused severe lung damage, and induced a robust pro-inflammatory response. IFN-α treatment effectively inhibited SARS-CoV-2 replication in the lung xenografts.

**Conclusions:** These data support the human lung xenograft mouse model as a useful and biological relevant tool that should facilitate studies on the pathogenesis of SARS-CoV-2 lung infection and the evaluation of potential antiviral therapies.

## Introduction

The coronavirus disease 2019 (COVID-19) pandemic, caused by severe acute respiratory syndrome coronavirus 2 (SARS-CoV-2) from *Coronoviridae* family, has become a serious public health issue globally 1-3. SARS-CoV-2 infection typically initiates in the upper respiratory tract and then progresses to the lower respiratory tract and other organs, producing a broad spectrum of clinical manifestations 4. Most patients suffered from COVID-19 pneumonia can potentially develop an acute respiratory distress syndrome (ARDS), which is associated with substantial morbidity and mortality 4. To develop effective strategies for prevention and treatment against COVID-19 pneumonia during the ongoing worldwide pandemic, characteristics and the pathogenesis of SARS-CoV-2 infection should be accurately understood in primary human target cells and tissues in a physiological setting, which highlights a pressing need for robust *in vitro* and *in vivo* human cell/tissue models mimicking natural SARS-CoV-2 infection.

Most recently, primary or induced pluripotent stem cell-derived human lung organoids have been widely used for studies of SARS-CoV-2-induced distal lung pathology 5-7. These models are made of human cells and can be rationally controlled to obtain the desired characteristics of lung tissues, and thus represent an appropriate physiologically relevant system for studies of SARS-CoV-2 infection and for screening of candidate COVID-19 therapeutics 5-9. However, these stem cell-based lung organoids still have some drawbacks, such as being technically tedious to establish 5-9, and the lack of complicated structures and compositions of the human lung, which consists of up to 40 different cell types 1, 2. These obstacles can be overcome by using a human-mouse chimeric lung mouse model, which is established by grafting human fetal lung tissues under the skin or renal capsules of immunodeficient mice 10-13. The chimeric humanized lung mouse model is technically easy to establish and has a key advantage that the grafted human fetal lung tissues typically vascularize, grow and develop mature structures closely resembling the normal adult human lung. To date, this *in vivo* model has been used to assess the replication and infectivity of many viruses, including Middle East respiratory syndrome coronavirus (MERS-CoV) 10, Zika virus 10, respiratory syncytial virus 10, human cytomegalovirus 10, 11 and varicella-zoster virus 13. This raises the question of whether SARS-CoV-2 replicates in the human lung xenografts and produces lung pathology which has features consistent with that seen in patients with COVID-19 pneumonia.

In this study, we provide, to our knowledge, the first characterization of SARS-CoV-2 infection dynamics and the ensuing inflammatory responses in a humanized lung mouse model. Our results show that SARS-CoV-2 efficiently replicated, caused lung damage and induced a pro-inflammatory response in human lung xenografts *in vivo*, which should provide insights into the pathogenesis and treatment of SARS-CoV-2 lung infection in humans.

## Methods

### Ethics and Biosafety statement

Human fetal lung tissues were obtained from spontaneous pregnancy losses (10 to 14 gestational weeks [g. w.]) at the Affiliated Zhongshan Hospital of Xiamen University. This study was approved by the Institutional Review Board of the hospital and the Research Ethics Committee of Xiamen University. Experiments were carried out in strict accordance with the guidelines and regulations of Xiamen University. Informed consent for use of the fetal tissues for research was obtained from the donating parents in accordance with the institutional guidelines. All experiments with infectious SARS-CoV-2 were performed in the biosafety level 3 (BSL-3) laboratory and animal biosafety level 3 (ABSL-3) laboratory facilities at the State Key Laboratory of Emerging Infectious Diseases, School of Public Health, The University of Hong Kong.

### Virus production, titer detection and storage

Viruses were propagated on Vero cells (ATCC, CCL-81) in Dulbecco's modified Eagle's medium (DMEM) containing 2% fetal bovine serum (FBS), 5 μg/mL TPCK-trypsin, and 30 mM MgCl_2_. Viruses were harvested and then titrated on Vero cells by TCID_50_ assay. Finally, the titers of viruses were adjusted to 2 × 10^4^ TCID_50_/mL (fourth-passage, 1 mL per stock) and stored at -80°C before use for animal study.

### Infection of human lung xenografts in SCID-hu mice

Human fetal lung tissues from a total of 5 donors were dissected into small fragments of ~10 mm^3^ in size. One piece of human lung tissue was engrafted subcutaneously into the right flank of each 4-6-week-old male C.B-17 severe combined immunodeficiency (SCID) mouse (Shanghai SLAC Laboratory Animal Technology Co., Ltd). At 8 weeks post-transplantation, lung xenografts were surgically exposed and mock-infected or infected with 1 × 10^3^ TCID_50_ SARS-CoV-2 in 50 μL volume with a 27-gauge needle. Lung xenografts were collected at 2, 4 and/or 6 days post infection (dpi) for subsequent analysis.

To explore the therapeutic effect of IFN-α on SARS-CoV-2 infection in human lung xenografts, two groups of lung xenografts were injected with 1 × 10^3^ TCID_50_ SARS-CoV-2 in 50 μL volume. One-hour after viral inoculation, a group of lung xenografts was treated with IFN-α2b (Xiamen Amoytop Biotech Co., Ltd., China) at a dosage of 3000 U in 100 μL volume, while the control group was treated with the same volume of saline. At 2, 4 and/or 6 dpi, lung xenografts were collected for subsequent analysis. All surgery was performed under isoflurane anesthesia, and all efforts were made to minimize animal suffering.

### Quantitative RT-PCR for SARS-CoV-2

Human lung xenografts were each weighed and cut into small pieces by sterile razor blade, and then homogenized in 1 mL sterile PBS with protease inhibitors using a loose Dounce homogenizer. Total RNA was extracted using a QIAamp Viral RNA Mini kit (Qiagen, 52904) according to the manufacturer's instructions. Real-time quantitative PCR was performed by the SLAN-96s Real-Time PCR System (Hongshi, China) with a Wantai SARS-CoV-2 RT-PCR Kit (Beijing Wantai Biological Pharmacy Enterprise Co., Ltd., China) according to the manufacturer's instructions.

### Histopathological analysis and immunohistochemistry

Paraffin sections (4 μm) of mock-infected and SARS-CoV-2-infected human lung xenografts were deparaffinized and rehydrated through a xylene and graded alcohol series. Next, sections were either stained with haematoxylin and eosin (H&E) for histological observation, or used for subsequent immunohistochemistry (IHC) analysis or for multiplex immunofluorescent assay.

IHC was performed as previously described 13. Briefly, following heat-induced antigen retrieval with 10 mM citrate buffer, sections were treated with 3% hydrogen peroxide to remove endogenous peroxidase and blocking reagent to block non-specific protein binding. Then, sections were incubated with primary mouse monoclonal antibodies of anti-SARS-CoV-2 N protein (clone 15A7, unpublished data), anti-human cytokeratin 19 (CK-19) (Abcam, ab7754) and anti-human vimentin (Abcam, ab8069) or rabbit polyclonal antibodies of anti-human angiotensin-converting enzyme 2 (ACE2) (Proteintech, 21115-1-AP), anti-human Clara cell 10 kDa protein (CC10) (Proteintech, 10490-1-AP), anti-human surfactant protein C (SP-C) (Proteintech, 10774-1-AP) and anti-human podoplanin (PDPN) (Proteintech, 11629-1-AP). Finally, IHC staining was performed using an Ultrasensitive TMS-P kit (Fuzhou Maixin Biotechnology Development Co., Ltd.) and a DAB detection kit (Fuzhou Maixin Biotechnology Development Co., Ltd.) according to the manufacturer's instructions. Sections were subsequently counterstained with hematoxylin, dehydrated and mounted on a slide and viewed under a Lecia DM4B microscope (Leica). IHC were performed with the technical support from Servicebio (Wuhan, China), Biofavor Biotech (Wuhan, China) and Xiamen Longshabio Technology (Xiamen, China). The airspace-tissue ratios and the percentage of SARS-CoV-2 N protein-positive areas were determined by means of established methods 14, 15. Ten fields of view per individual lung xenograft were assessed at 400× magnification with the use of ImageJ software (MA, USA).

Multiplex fluorescence labeling was performed on the sections using Opal^TM^ 7-Color Manual IHC Kit (PerkinElmer, NEL811001KT) following heat-induced antigen retrieval, non-specific protein blocking and endogenous peroxidase blocking as described above. Briefly, sections were incubated with primary antibodies described above at room temperature, followed by detection using the HRP-conjugated secondary antibodies (1:1000) and TSA-dendron-fluorophores (1:100). Then, the primary and secondary antibodies were eliminated by heating the slides in retrieval buffer using microwave. In a serial fashion, each antigen was labeled with a distinct fluorophore dye. Multiplex antibodies applied in this study include two panels. The first panel includes anti-human ACE2 (1:10000), anti-SARS-CoV-2 N protein (1:5000), anti-human vimentin (1:20000) and anti-human CK-19 (1:10000). The second panel includes anti-human ACE2 (1:10000), anti-SARS-CoV-2 N protein (1:5000), anti-human PDPN (1:20000) and anti-human SP-C (1:1000). After all the antibodies were detected sequentially, nuclei were stained with DAPI (Thermo Fisher, D1306) and the slides were imaged using the White Light Laser Confocal Microscope Leica TCS SP8 X (Leica). Multiplex immunofluorescence assay was performed with the technical support from Biofavor Biotech (Wuhan, China) and Xiamen Longshabio Technology (Xiamen, China).

### Measurement of cytokines and chemokines in human lung xenografts

Supernatants of human fetal lung tissues homogenates were used for 20 specific human cytokines/chemokines measurement using ELISA kits (Elabscience) according to the manufacturer's instructions.

### Statistical analysis

The expression level of cytokine and chemokines were calculated by using Origin software (Origin 2019b). Statistical analyses were carried out using Prism software (GraphPad Prism 7.0), and results were presented as mean ± standard error of mean (SEM) and single comparisons were made using unpaired two-tailed Student's t-tests. Statistical details of experiments and animal replication numbers (n) are stated in the relevant figure legends and method details. A *p* value less than 0.05 is considered statistically significant.

## Results

### SARS-CoV-2 replication in human lung xenografts

**Figure 1:**
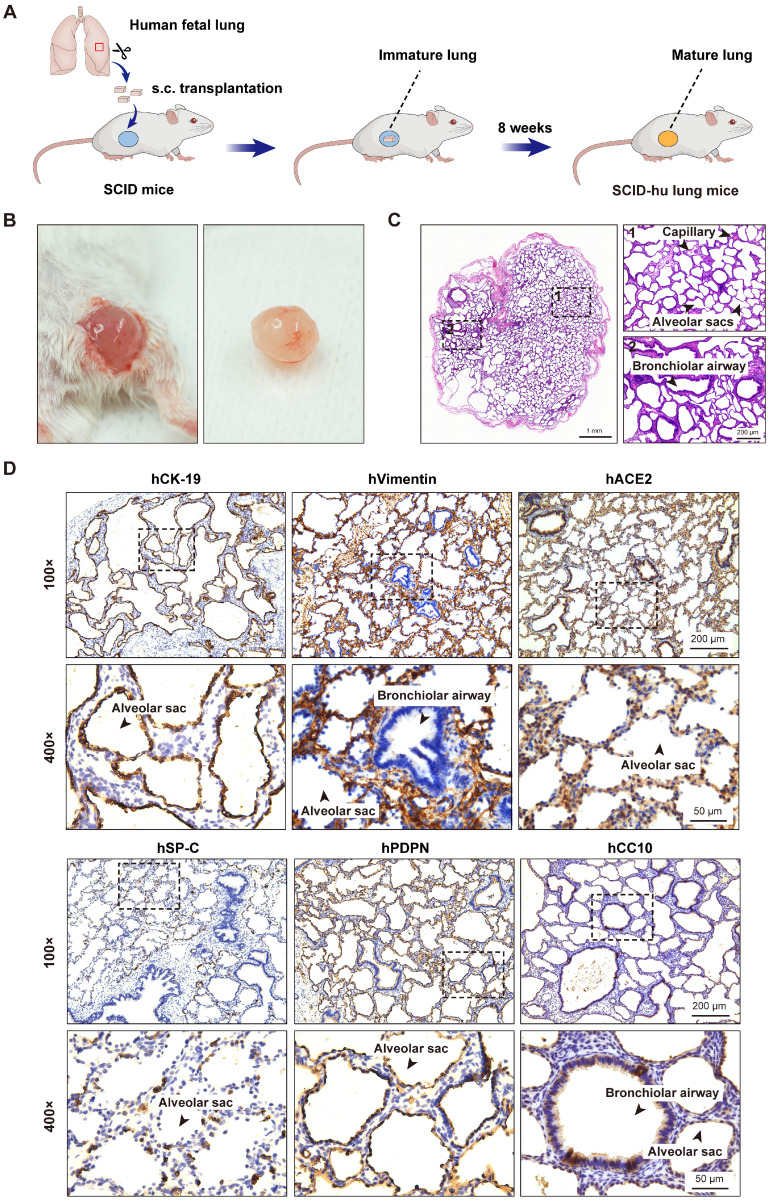
** Subcutaneous transplantation of human fetal lung tissues into SCID mice to develop the human lung xenograft mouse model. (A)** Scheme of construction of the human lung xenograft mouse model. Small fragments (~10 mm^3^) of human fetal lung tissues were transplanted subcutaneously (s.c.) into the right flank of SCID mice. After 8 weeks, the lung xenografts can grow and develop mature lung structures. **(B)** Gross view and **(C)** histological analysis of the human lung xenograft at 8 weeks post-transplantation. (D) Immunohistochemistry staining of lung marker expression in the human lung xenograft. Sections of human lung xenografts at 8 weeks post-transplantation were immunostained for human CK-19 (hCK-19), human vimentin (hVimentin), human PDPN (hPDPN), human SP-C (hSP-C), human CC10 (hCC10) to visualize the lung epithelium and mesenchyme, type I and type II pneumocytes, and bronchiolar Clara cells, respectively. Tissue sections were also stained for human ACE2 (hACE2) expression. The lower panels represent higher magnification images of the areas outlined by the dotted lines in the upper panels. Scale bars in the left and right panels in **(C)** represent 1 mm and 200 µm, respectively. Scale bars for 100 × and 400 × magnification in **(D)** represent 200 µm and 50 µm, respectively. Staining was performed on tissue sections from 4 lung xenografts obtained from two donors, and representative images are shown.

To generate the SCID-hu lung mouse model, one small piece of human fetal lung tissue (~10 mm^3^) was grafted subcutaneously into the right flank of each SCID mouse. After 8 weeks, the fetal lung xenografts increased to a mean volume of 0.82 cm^3^ (±0.45 cm^3^) in size. Surgical excision and histological examination of these human lung xenografts revealed prominent airspace dilation, well-defined vasculature and well-developed bronchioles and alveolar saccules (Figure 1A-C). Further immunohistochemical analysis revealed the presence of human epithelial and mesenchymal cells staining positive for CK-19 and vimentin, respectively. The presence of alveolar type 1 (AT1, positive for PDPN) and type 2 (AT2, positive for SP-C) cells in the alveolar epithelium, as well as Clara cells (positive for CC10) in the bronchiolar epithelium was also confirmed (Figure 1D). Furthermore, a positive staining for the cellular receptor for SARS-CoV-2 virus, human ACE2 was found mainly in the lung epithelium (Figure 1D). Overall, human fetal lung tissues grafted under the skin of SCID mice were well-vascularized, well-developed with multiple lung cell types differentiated, and easily accessible to virus inoculation.

**Figure 2:**
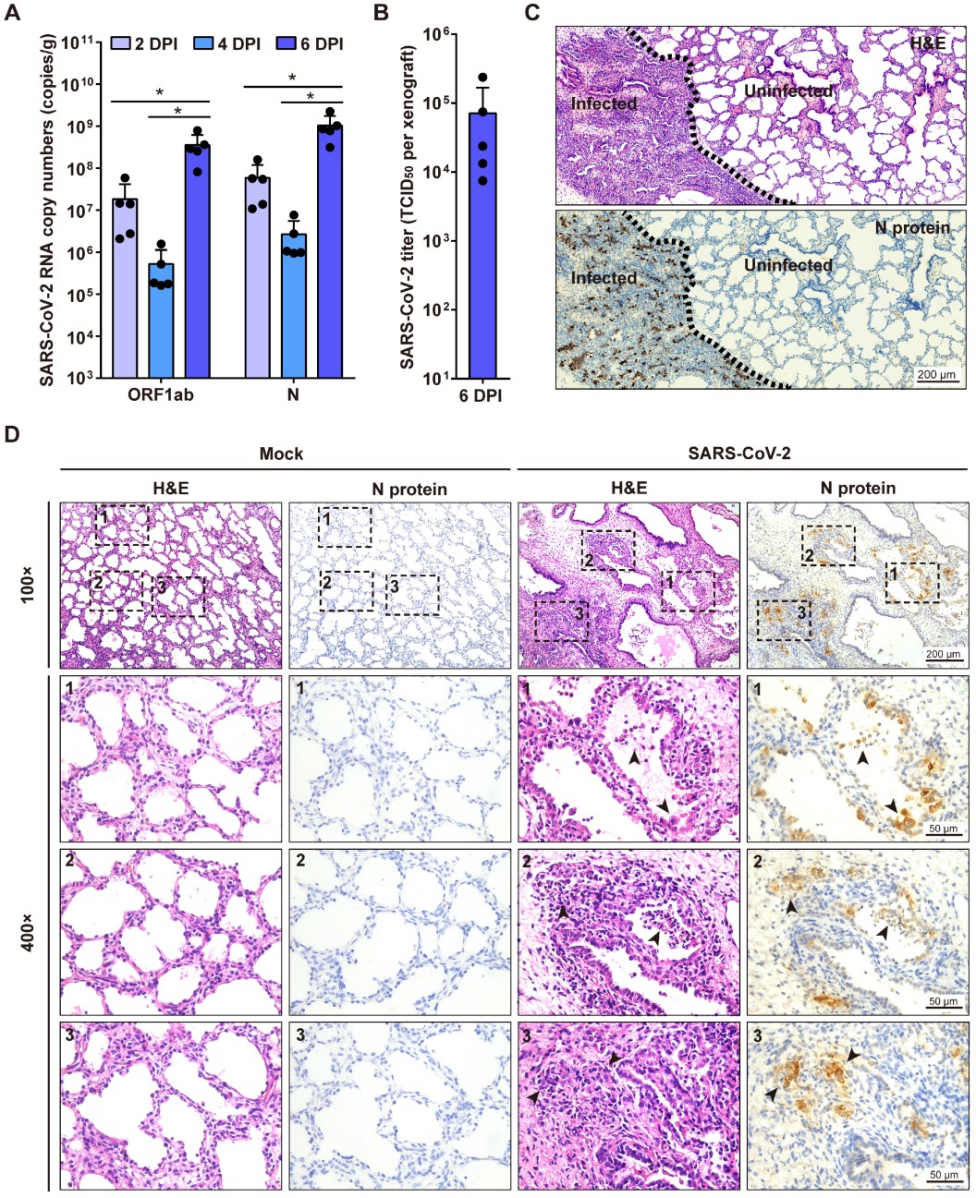
** SARS-CoV-2 infection of human lung xenografts in SCID-hu mice. (A)** SARS-CoV-2 genome copy numbers in the infected human lung xenografts harvested at 2, 4 and 6 dpi (n = 5 mice per group per time point). (**B**) Infectious virus yields in SARS-CoV-2-infected human lung xenografts harvested at 6 dpi (n = 5). Error bars denote SEM, and asterisks denote significant differences (*P<0.05; unpaired two-tailed Student's t test compared to the controls). **(C** and** D)** H&E staining and IHC analysis were employed for histopathological evaluations of the SARS-CoV-2 infected human lung xenografts harvested at 6 dpi. Dotted lines in **(C)** separate the SARS-CoV-2-infected lung lesions from the uninfected lung tissue. Compared with the mock-infected lung tissue, obvious histopathological changes have been observed in SARS-CoV-2-infected regions, as indicated by black arrows in **(D)**, which showed desquamated infected epithelial cells, consolidation, thickness of alveolar walls, collapsed alveolar spaces and the loss of airway architecture, etc. The lower panels (400 × magnification) in **(D)** represent higher magnification images of the areas outlined by the dotted lines in the upper panels (100 × magnification). Scale bar in **(C)** represents 200 µm. Scale bars for 100 × and 400 × magnification in **(D)** represent 200 µm and 50 µm, respectively. Staining was performed on tissue sections from two lung xenografts per group, which were obtained from two donors, and representative images are shown.

Next, we investigated SARS-CoV-2 replication in human lung xenografts at 8 weeks post-transplantation. Human lung xenografts were mock-infected or infected with 1 × 10^3^ TCID_50_ SARS-CoV-2 (50 μL, 27-gauge needle) (n = 5 in each group). Then viral RNA loads in tissue homogenates of each human lung xenograft were determined by quantitative RT-PCR (qRT-PCR) probing for both N gene and ORF1ab gene at 2, 4 and 6 dpi. After inoculation, the genome copy numbers in the infected lung xenografts significantly increased to 10^8^ to 10^9^ copies/g at day 6 compared to day 2 and day 4 (Figure 2A). Furthermore, infectious viruses were isolated from a group of infected lung xenografts (n = 5) at 6 dpi, and the mean titer was 7.1 × 10^4^ TCID_50_ per xenograft (Figure 2B). These results suggest active SARS-CoV-2 replication in human lung xenografts. No mice showed body weight loss throughout the experiment.

### SARS-CoV-2-induced histopathology in human lung xenografts

To examine the histopathological changes associated with SARS-CoV-2 infection in the human lung xenografts, tissue sections were stained with H&E and also immunostained for viral N protein expression compared with the mock-infected controls. At 6 dpi, positive staining for the SARS-CoV-2 N protein was readily observed throughout the infected lung tissues (Figure 2C-D) and accounted for a mean of 15.4% of tissue areas by IHC image quantification. Notably, SARS-CoV-2 infection induced severe histopathological alterations in the lung xenografts. In SARS-CoV-2-infected lung lesions, desquamated infected epithelial cells were found in the airway occasionally. Furthermore, there were substantial areas with hemorrhage, consolidation, thickness of alveolar walls, collapsed alveolar spaces and loss of architecture of the small airways (Figure 2C-D). In contrast, no N protein staining was observed, and tissue structures remained intact in uninfected areas and the mock-infected lung xenografts (Figure 2C-D). By quantification, the mean airspace-tissue ratios of the mock and infected lung xenografts were 55.63% and 38.27%, respectively, indicating destruction of lung airspace by SARS-CoV-2 infection. Additionally, direct intra-xenograft injection of SARS-CoV-2 into the human lung xenografts did not result in histopathological changes in any visceral tissue of SCID mice (data not shown).

### SARS-CoV-2 tropism in human lung xenografts

**Figure 3:**
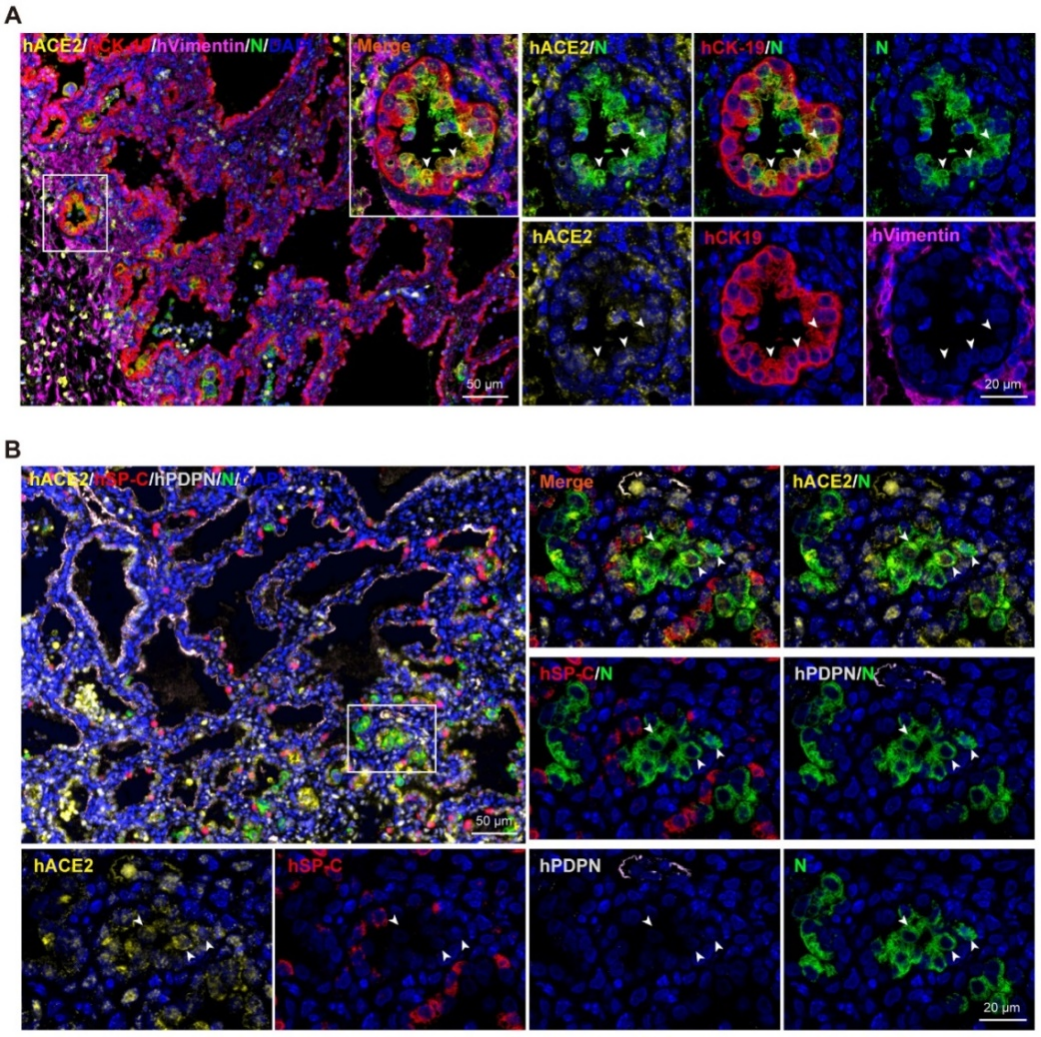
** SARS-CoV-2 tropism in human lung xenografts.** Sections of SARS-CoV-2-infected lung xenografts harvested at 6 dpi were subjected to multiplex immunofluorescence assay for **(A)** co-staining of SARS-CoV-2 N protein (N, green), human ACE2 (hACE2, yellow), human CK-19 (hCK-19, red) and human vimentin (hVimentin, magenta), or for **(B)** co-staining of SARS-CoV-2 N protein (green), human ACE2 (hACE2, yellow), human SP-C (hSP-C, red) and human PDPN (hPDPN, grey). Nuclei were stained with DAPI (blue). White frame was magnified on the right and bottom panels. Solid white arrows indicate the SARS-CoV-2+/hACE2+ cells. Scale bars in the upper left panels in **(A)** and** (B)** represent 50 µm, while those in other panels represent 20 µm. Staining was performed on tissue sections from two lung xenografts obtained from two donors, and representative images are shown.

To identify the cells targeted by SARS-CoV-2 in our model, sections from SARS-CoV-2-infected lung xenografts collected at 6 dpi were further stained using multiplex immunofluorescence assay for different cell markers, including CK-19 for lung epithelial cells, Vimentin for lung mesenchymal cells, PDPN for AT1, SP-C for AT2, as well as viral N protein and hACE2. As shown in Figure 3A-B, expression of SARS-CoV-2 N protein coincided with the areas of histopathological changes at 6 dpi and predominantly co-localized with hCK-19 and hACE2, indicating that the hACE2-expressing lung epithelial cells are the major target cells of SARS-CoV-2 in the human lung xenografts. Notably, decreased or loss of expression of hACE2 and lung surfactant proteins was observed in SARS-CoV-2-infected cells, which is consistent with recent findings in human distal lung organoids and in COVID-19 patients 6, 16, 17.

### Inflammatory responses to SARS-CoV-2 infection in human lung xenografts

**Figure 4:**
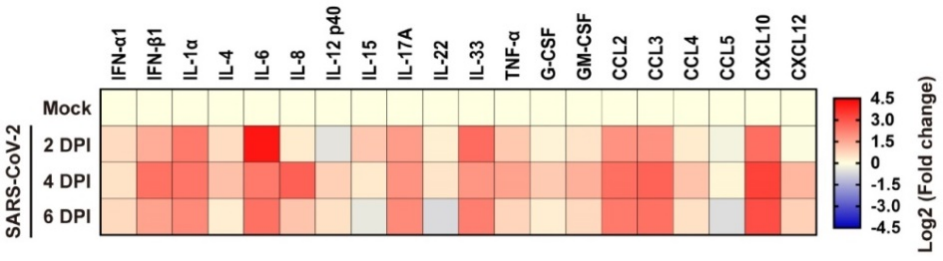
**Cytokine/chemokine response to SARS-CoV-2 infection in human lung xenografts.** A total of 20 cytokines/chemokines were measured in SARS-CoV-2 infected human lung xenografts at 2, 4 and 6 dpi. For each cytokine/chemokine, the fold change was calculated as compared with mock-infected xenografts and the log_2_ (fold change) was plotted in the corresponding heat map. Two independent experiments were performed with n = 5 mice per group per time point. Human lung xenografts used in these experiments were obtained from two donors.

To elucidate the inflammatory responses in human lung xenografts to SARS-CoV-2 infection, expression of 20 kinds of human cytokines/chemokines in homogenates of mock-infected and SARS-CoV-2-infected lung xenografts (n = 5 in each group) were determined by ELISA at 2, 4 and 6 dpi. Compared with the mock-infected controls, SARS-CoV-2 infection in the human lung xenografts resulted in upregulated expression of most pro-inflammatory cytokines and chemokines, including IFN-α1, IFN-β1, IL-1α, IL-4, IL-6, IL-8, IL-12 p40, IL-15, IL-17A, IL-33, TNF-α, G-CSF, GM-CSF, CCL2/MCP-1, CCL3/MIP-1α, CCL4/MIP-1β, CXCL10/IP-10 and CXCL12 (Figure 4). Expression kinetics of these cytokines/chemokines varied over the 6-day period. The levels of IFN-β1, IL-1α, IL-6, IL-17A, IL-33, CCL2, CCL3 and CXCL10 increased substantially and maintained at relatively high levels throughout the experiment. IL-8 reached high peak values at 4 dpi and then declined to control level, while elevations of other cytokines or chemokines were mild to moderate.

### IFN-α antiviral effects against SARS-CoV-2 infection in human lung xenografts

**Figure 5:**
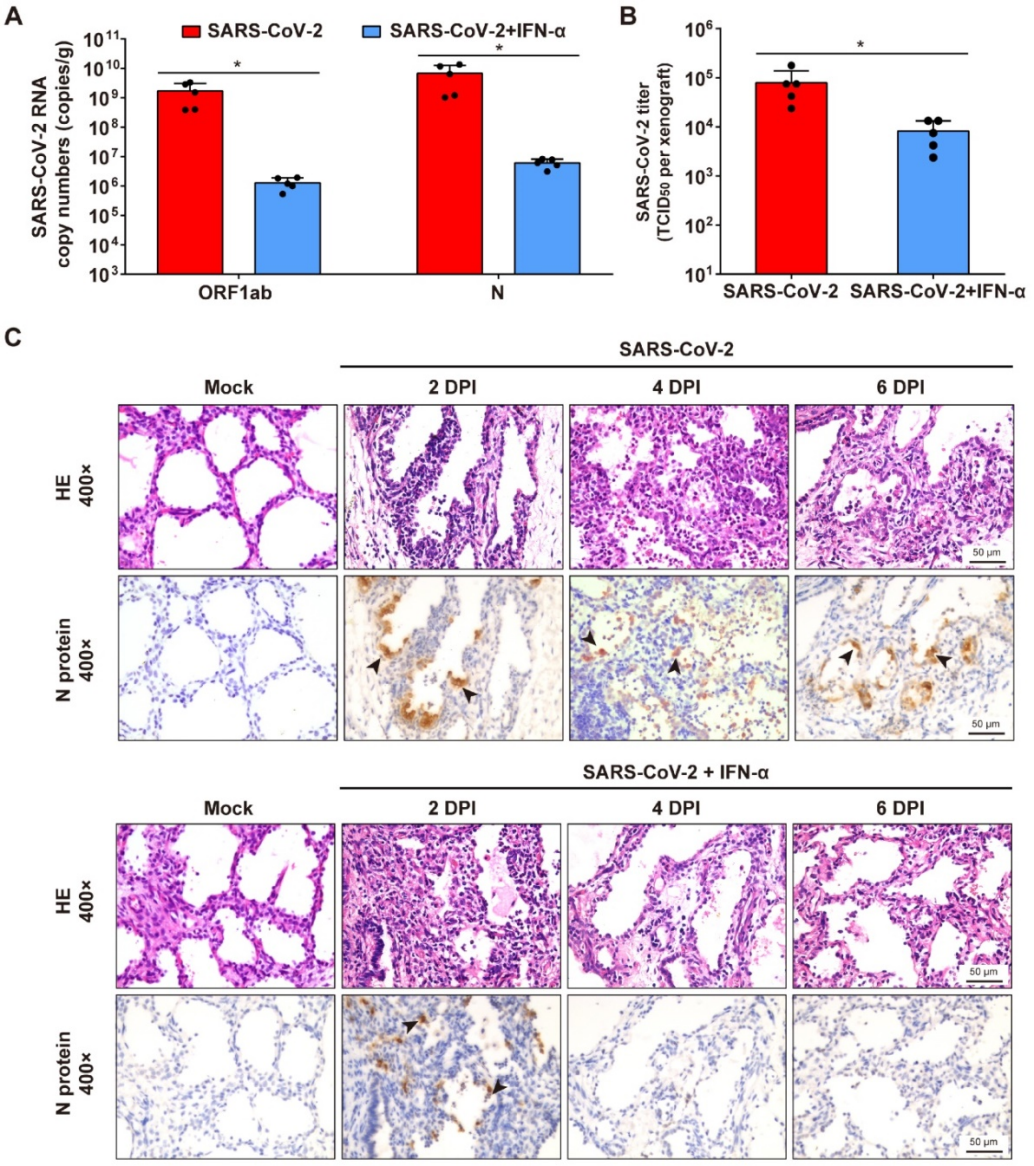
** IFN-α inhibits SARS-CoV-2 replication in human lung xenografts.** Human lung xenografts in SCID-hu mice were inoculated with 1 × 10^3^ TCID_50_ SARS-CoV-2 1 h before treatment with 3000 U of IFN-α or saline as a control (each n = 5). **(A)** SARS-CoV-2 genome copy numbers and **(B)** infectious virus yields were determined at 6 dpi. Error bars denote SEM, and asterisks denote significant differences (*P<0.05; unpaired two-tailed Student's t test compared to the controls). **(C)** Histological analysis and IHC staining for SARS-CoV-2 N protein expression in human lung xenografts were performed at 2, 4 and 6 dpi. Solid black arrows indicate the SARS-CoV-2-infected cells. Scale bars represent 50 µm. Staining was performed on tissue sections from two lung xenografts per group, which were obtained from two donor and representative images are shown.

To determine whether interferon-α (IFN-α) have protective effects on SARS-CoV-2 infection of human lung tissues, each lung xenograft was treated with 3000 U of IFN-α (100 μL, 27-gauge needle) or equal volume of saline 1 h after inoculation with 1 × 10^3^ TCID_50_ of SARS-CoV-2. The results showed that this IFN-α treatment effectively inhibited viral RNA replication and infectious virus production at 6 dpi (Figure 5A-B). Subsequent immunostaining for viral protein expression and histopathology analysis showed that viral translation was observed in several areas of the alveolar epithelium of the IFN-treated infected lung xenografts at 2 dpi, but neither viral protein nor any histopathological change was detected in xenografts at 4 and 6 dpi (Figure 5C), indicating that there likely was an initial cycle of replication before the infection was controlled by the IFN-α antiviral activity.

## Discussion

SARS-CoV-2, along with two other closely related coronaviruses, SARS-CoV and MERS-CoV, is a highly pathogenic virus that can cause severe life-threatening pneumonia in affected individuals 4. A hallmark of severe COVID-19 pneumonia is SARS-CoV-2 infection of the epithelial cells of the distal lung 18-20. However, limited access to these cells from patients hampers progress in understanding of the initial responses of human lung cells to SARS-CoV-2 infection in the physiological state. To address this limitation, the present study evaluated SARS-CoV-2 infection in a SCID mouse-human lung xenograft model *in vivo*. Human fetal lung tissues grafted under the dorsal skin of SCID mice developed mature structures and multiple lung cell types. Thus, this model provides an *in vivo* environment closely resembling normal human adult lung tissues and should be favorable for studying SARS-CoV-2 lung infection. Indeed, human lung xenografts in the SCID-hu mice were highly permissive for SARS-CoV-2 infection, and exhibited substantial viral RNA replication, viral protein expression and production of infectious viral particles post-infection. The lung epithelial cells seem to be the main target for SARS-CoV-2 infection. Furthermore, SARS-CoV-2 infection resulted in a robust local pro-inflammatory response in human lung xenografts. The pattern of upregulation of cytokines/chemokines, like IL-6, TNF-α, IL-12, IFN-β and IL-17A and chemokines GM-CSF, CCL2, CCL3 and CXCL10, was similar to those observed in COVID-19 patients 21, 22. These data suggest that this human lung xenograft mouse model provides a useful, biologically relevant tool to investigate human lung infection by SARS-CoV-2.

The SCID-hu lung mouse model can serve as an easy alternative approach and a complement to the current widely used human lung organoid model. Both models have been used to simulate accurate lung cellular responses to perturbations like viral infections in humans 5-7, 10, 12, 13, 23. The human lung organoid model typically mimics the alveolar epithelium and can be long-term propagated* in vitro*. However, this model lacks complete cellular complexity of human lung tissues and requires highly stringent culture conditions and tedious experimental procedures 5-8, 23. On the other hand, *in vivo* human lung xenografts develop mature structures of adult human lung and is technically easy to generate in large numbers. These two models can reveal response characteristics of human primary lung to SARS-CoV-2 infection at the cellular and tissue levels, respectively, and should be used together to better meet the current urgent need for research in this field.

The SCID-hu lung mouse model has the potential for evaluating antiviral therapies against SARS-CoV-2 infection *in vivo*. Here we preliminarily tested IFN-α treatment for protecting human lung xenografts from SARS-CoV-2 infection. Type I IFN signaling is important for generation of antiviral innate and adaptive immunity 24, 25. Although literatures have shown that type I IFN deficiency characterizes severe or critical COVID-19 cases, the potential for response to type I IFN is not affected in COVID-19 patients regardless of disease severity 26. Therefore, type I IFN administration could be of therapeutic benefit in the treatment of COVID-19. Recent studies have shown IFN-β treatment effectively blocks SARS-CoV-2 replication in cell culture 26, 27, while our study showed that IFN-α treatment also efficiently controlled SARS-CoV-2 replication in human lung xenografts *in vivo*. These experimental data are consistent with recent clinical reports showing that early use of type I IFN can reduce mortality of COVID-19 patients 28. Together, these findings should support the validity of type I IFN for COVID-19 treatment.

In addition, optimal models could be achieved by generating humanized mice with not only human functional lungs but also human immune system to more closely recapitulate immunopathology during COVID-19 pneumonia in the future 10, 29. Further studies using these chimeric humanized lung mouse models should help identify viral or host factors to improve understanding of molecular mechanisms of SARS-CoV-2 lung pathogenesis and aid in identifying potential therapeutic targets for treatment of SARS-CoV-2-associated lung diseases.

## Abbreviations

ACE2: angiotensin-converting enzyme 2
ARDS: acute respiratory distress syndrome
AT: alveolar type
CCL: C-C motif chemokine ligand
CK-19: cytokeratin 19
COVID-19: coronavirus disease 2019
CXCL: C-X-C motif chemokine ligand
Dpi: days post infection
G-CSF: granulocyte colony-stimulating factor
GM-CSF: granulocyte-macrophage colony stimulating factor
H&E: haematoxylin and eosin
IFN: interferon
IHC: immunohistochemistry
IL: interleukin
IP: interferon gamma-induced protein
MCP: monocyte chemotactic protein
MERS-CoV: Middle East respiratory syndrome coronavirus
MIP: macrophage inflammatory protein
PDPN: podoplanin
SARS-CoV: severe acute respiratory syndrome coronavirus
SCID: severe combined immunodeficiency
SP-C: surfactant protein
TCID_50_: median tissue culture infective dose
TNF: tumor necrosis factor
